# A Retrospective Study on Drug and Dietary Patterns in Patients With Severe Rheumatic Valvular Heart Disease in Eastern India

**DOI:** 10.7759/cureus.37358

**Published:** 2023-04-10

**Authors:** Debasish Das, Satyapriya Mohanty, Satyabrata Guru, Anindya Banerjee, Abhinav Kumar, Pranjit Deb, Humshika Samantray

**Affiliations:** 1 Cardiology, All India Institute of Medical Sciences, Bhubaneswar, IND; 2 Cardiothoracic and Vascular Surgery, All India Institute of Medical Sciences, Bhubaneswar, IND; 3 Trauma and Emergency/Internal Medicine, All India Institute of Medical Sciences, Bhubaneswar, IND; 4 Cardiothoracic Surgery, All India Institute of Medical Sciences, Bhubaneswar, IND

**Keywords:** retrospective study, eastern india, rheumatic valvular heart disease, dietary pattern, drug

## Abstract

Background

India has a high prevalence of rheumatic valvular heart diseases. Empirical treatment in rheumatic heart disease curtails morbidity and mortality. Less is known about the drug and dietary management of severe rheumatic heart disease at the pretertiary care level, which forms the building stone in the management of rheumatic heart disease. The present study was carried out to evaluate the drug and dietary patterns of patients with severe rheumatic valvular heart disease at a pretertiary care level, which is the backbone of the management of rheumatic heart disease.

Methodology

This cross-sectional study was carried out in a tertiary care center in Eastern India between May 2020 and May 2022 across 1,264 study subjects. The drug and dietary patterns of the patients with severe rheumatic valvular heart disease during their index visit to the cardiac department were studied and analyzed. Patients aged less than 18 years; patients with mild or moderate rheumatic valvular heart diseases; patients with coexisting end-stage organ disease (chronic liver disease and chronic kidney disease), malignancy, and sepsis; and patients not willing to participate in the study were excluded.

Results

Most of the patients were on diuretic therapy, and diuretic therapy was overprescribed across the patients with mitral regurgitation, aortic stenosis, and aortic regurgitation. Most of the patients across each spectrum of rheumatic valvular heart disease were lacking the cornerstone therapy such as beta-blockers in mitral stenosis and angiotensin-converting enzyme (ACE) inhibitors or angiotensin receptor blockers (ARBs) in mitral and aortic regurgitation. The recommended injectable benzathine penicillin prophylaxis was prescribed in a very small number of patients (5%), and most of the patients were on oral penicillin prophylaxis (95%) in spite of its reported high failure rate in prophylaxis. Empirical rationale prescriptions in severe rheumatic valvular heart disease were lacking in the pretertiary care level in Eastern India.

Conclusion

Each spectrum of severe valvular heart disease was lacking the cornerstone therapy such as beta-blockers in mitral stenosis and ACE inhibitors or angiotensin receptor blockers (ARBs) in mitral and aortic regurgitation along with recommended injectable benzathine penicillin prophylaxis. Diuretics and digoxin were overprescribed across the spectrum of rheumatic heart disease. Improvement of this essential gap in the treatment of severe rheumatic heart disease would bring down morbidity and improve mortality in the future.

## Introduction

Drugs and diet play pivotal roles in rheumatic heart disease. It is often noted in clinical practice that patients with rheumatic heart disease are undertreated by practicing physicians. Most often, they are prescribed only diuretics and, in some cases, digoxin, even if the patient is not in atrial fibrillation or heart failure [1], which may be associated with increased mortality. Patients with regurgitant lesions are often not advised vasodilators such as angiotensin-converting enzyme (ACE) inhibitors or angiotensin receptor blockers (ARBs) as echocardiography is not available in rural areas to reach a definite cardiac diagnosis besides other factors such as lack of knowledge and awareness among attending physicians. Many patients with rheumatic heart disease are prescribed oral penicillin prophylaxis in spite of its reported high failure rate [2]. Most often, patients with rheumatic heart disease are not advised of a proper anticoagulation regimen even if they are in atrial fibrillation. There exists a definite gap between the recommended medical management and actual practice of rheumatic heart disease by primary care physicians. The present study aims to evaluate the real-world scenario demonstrating the gap between recommendation and actual medical practice in rheumatic heart disease. The present study was intended to analyze the drug and dietary patterns of patients with rheumatic heart disease at a pretertiary care level during their index visit to the cardiac department in a tertiary care hospital in Eastern India so that knowledge of the same would enrich the level of patient care in rheumatic heart disease.

## Materials and methods

Aim and objective

We aim to retrospectively study and analyze the drug and dietary patterns of patients with severe rheumatic valvular heart disease in a tertiary care center in Eastern India.

Inclusion criteria

Patients aged more than 18 years and those with severe rheumatic valvular heart disease of any single valve were included. We had taken into account the predominant type of valvular lesion across mixed or combined lesions to decide on any of the four categories to delineate the proper therapeutic measure: mitral stenosis, mitral regurgitation, aortic stenosis, and aortic regurgitation.

Exclusion criteria

We excluded those less than 18 years old; patients with mild or moderate rheumatic valvular heart diseases, end-stage organ disease (chronic liver disease and chronic kidney disease), malignancy, and sepsis; and those not willing to participate in the study.

The present retrospective study was conducted in a tertiary care center in Eastern India from May 2020 to May 2022. Sample size calculation was done using the standard formula with the power of the study being 80% with an attrition rate of 15%. After obtaining institutional ethical clearance for retrospective analysis, we analyzed the prescription data of 1,264 patients attending the outpatient department of cardiology and cardiothoracic and vascular surgery between May 2020 and May 2022 during their index visit to the tertiary care center. Informed patient consent was obtained from each of those patients. A brief history of the demographic details and clinical profile of each patient were obtained. They were divided into four strata of patients with mitral stenosis, mitral regurgitation, aortic stenosis, and aortic regurgitation. The drug and dietary patterns of each patient during their index visit to the tertiary care hospital were noted and analyzed. The dietary pattern of the patients included salt and water restriction and selective food restriction while taking anticoagulants.

Data were entered in an Excel sheet (Microsoft Corp., Redmond, WA, USA) in tabular form. All nominal and ordinal variables were represented in tabular form, and descriptive statistics were used to analyze the data using the Statistical Package for the Social Sciences (SPSS) software version 21 (IBM SPSS Statistics, Armonk, NY, USA). The chi-square test was used for tests of statistical significance, wherever appropriate, and a P value < 0.05 was considered statistically significant.

## Results

Table 1 reflects the mean age of our study population of 35.68±10.82 years. Females constituted 60% of the study population, whereas males constituted 40% of the study population. Of the patients with rheumatic heart disease, 25% drink alcohol. More than one-third of the study population were smokers, and the majority of the male rheumatic heart disease patients were smokers (35%). A family history of sudden cardiac death was present in 2% of the patients with rheumatic heart disease.

**Table 1:** Baseline variables of the study subjects SD: standard deviation

Variables	Total number of patients (%) (N=1,264)
Age (mean±SD) in years	35.68±10.82
<30 years	379 (30)
30-40 years	632 (50)
40-50 years	127 (10)
50-60 years	63 (5)
>60 years	63 (5)
Male	506 (40)
Female	758 (60)
Obesity	101 (8)
Alcohol intake	316 (25)
Smoking	442 (35)
Dyslipidemia	126 (10)
Family history of sudden cardiac death	25 (2)

**Figure 1:**
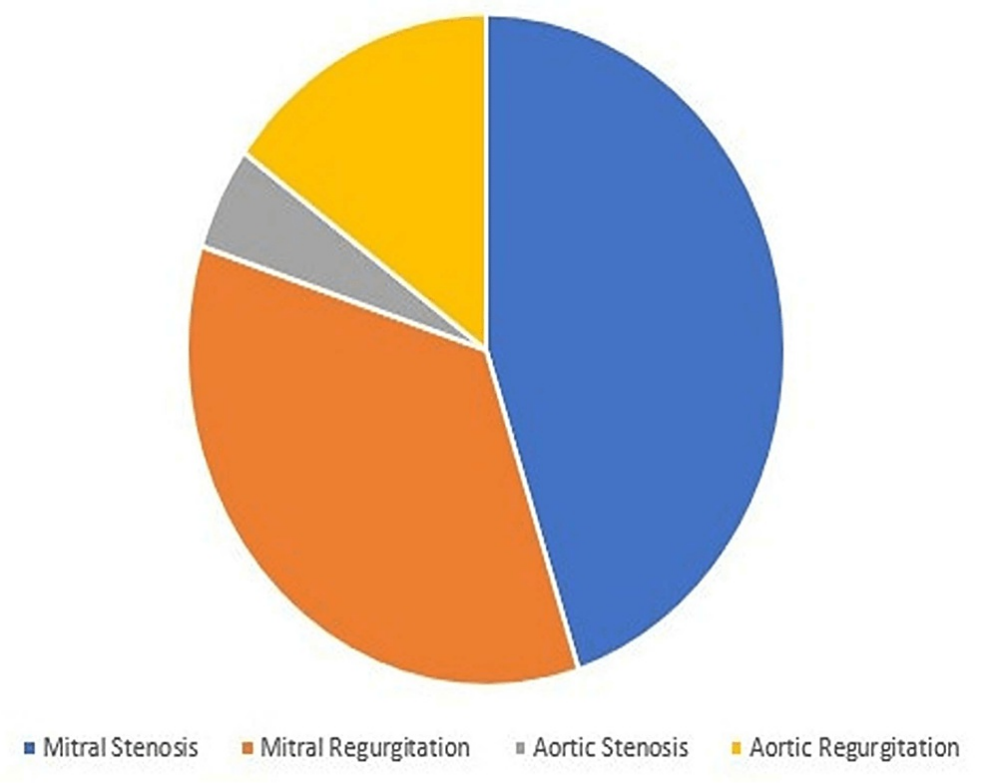
Type of predominant valvular lesion across the study subjects

Table 2 and Figure 1 reflect the pattern of predominant valvular lesions across the study subjects. Mitral stenosis was the predominant lesion in the majority (45%) of cases, followed by mitral regurgitation (35%). Predominant aortic regurgitation was noted in 15% of cases in whom there was combined involvement of the mitral valve and aortic valve with thickening of both anterior and posterior mitral leaflets. Predominant rheumatic aortic stenosis was noted in a smaller number of cases (only 5%), where the aortic valve was tricuspid in echocardiography with thickening of the mitral valve as a surrogate marker of rheumatic etiology. About 60% (758 patients) of cases had combined valvular heart disease in the form of mitral stenosis with mitral regurgitation (40% or 505 patients) or mitral stenosis with aortic regurgitation (20% or 253 patients). We had taken into account the predominant type of valvular lesion across mixed or combined lesions to decide on any of the above four categories to delineate the proper therapeutic measure.

**Table 2:** Type of valvular lesion across the study subjects

Pattern of predominant valvular involvement	Total number of cases (percentage) (N=1,264)
Mitral stenosis	568 (45)
Mitral regurgitation	442 (35)
Aortic stenosis	63 (5)
Aortic regurgitation	191 (15)

Table 3 reflects the drug and dietary patterns of the patients with rheumatic heart disease. Most of the patients with rheumatic heart disease (90%) were on diuretics. The cornerstone of management of patients with mitral stenosis is beta-blockers, which most patients (90%) were not receiving. The most commonly prescribed drug in peripheral practice is digoxin, which was administered in 40% of cases. Only 10% of cases of rheumatic mitral stenosis were receiving anticoagulants. Only 30% of patients with rheumatic mitral stenosis were following salt restriction, and fluid restriction of less than 1.5 L/day was practiced among 5% of patients. One interesting finding was that only 15% of patients with severe mitral regurgitation were receiving ACE inhibitors. Of aortic stenosis patients who are prone to suffer from low cardiac output and hypotension, 60% were receiving full-dose diuretics. Beta-blocker was administered in 35% of patients with severe aortic stenosis, which deleteriously affects severe aortic stenosis by decreasing the heart rate and force of contraction of the heart, thereby decreasing the net forward cardiac output, making those patients more prone to suffer low cardiac output symptoms in the form of hypotension, presyncope, and syncope. Antiplatelet and statin were administered in only 10% of cases of aortic stenosis. ACE inhibitors and ARBs are considered the cornerstone of the management of aortic regurgitation. However, ARBs were administered in only 20% of cases of aortic regurgitation. In contrast, beta-blockers were administered in 45% of cases of aortic regurgitation for palpitation in those patients, which is not a true arrhythmic palpitation but a thumping sensation in the heart secondary to volume overload. Most of the patients with aortic regurgitation (80%) received diuretics. Most of the patients with rheumatic heart disease (95%) were on oral penicillin prophylaxis, and only 5% of the cases were on recommended injectable benzathine penicillin prophylaxis.

**Table 3:** Drug and dietary patterns of patients with RHD RHD: rheumatic heart disease, LV: left ventricular, EF: ejection fraction, AF: atrial fibrillation, ACE: angiotensin-converting enzyme, ARBs: angiotensin receptor blockers

Type of valvular lesion	LV systolic dysfunction (EF<50%) (number of cases (%))	Drugs and diet therapy	Number of cases (%)
Mitral stenosis	29 (5)	Diuretics	511 (90)
		Beta-blockers	57 (10)
		Digoxin	227 (40)
		Anticoagulants for AF	57 (10)
		Calcium channel blocker (verapamil)	29 (5)
		Salt restriction	170 (30)
		Fluid restriction of <1.5 L/day	29 (5)
Mitral regurgitation	88 (20)	ACE inhibitors	66 (15)
		ARBs	22 (5)
		Diuretics	375 (85)
Aortic stenosis	6 (10)	Beta-blocker	22 (35)
		Digoxin	3 (5)
		Full-dose diuretics	39 (60)
		Antiplatelets	6 (10)
		Statins	6 (10)
Aortic regurgitation	9 (5)	ACE inhibitors	9 (5)
		ARBs	38 (20)
		Beta-blockers	86 (45)
		Diuretics	153 (80)
All spectra of RHD		Oral penicillin	1,201 (95)
		Injectable benzathine penicillin	63 (5)

The drug intake across the stenotic and regurgitant lesions was compared (Table 4). It was observed that there was a significant difference in the use of beta-blockers across the patients with aortic stenosis and mitral stenosis: in patients with mitral stenosis, beta-blocker was underprescribed, and in patients with aortic stenosis, beta-blocker was overprescribed. Digoxin was overprescribed in patients with mitral stenosis (P<0.0001) without the presence of atrial fibrillation. ACE inhibitors, ARBs, and diuretics were similarly used in patients with mitral and aortic regurgitation (P>0.05).

**Table 4:** Comparison of drug intake across stenotic and regurgitant lesions ACE: angiotensin-converting enzyme, ARBs: angiotensin receptor blockers

Type of drug	Mitral stenosis (n=568) (number (%))	Aortic stenosis (n=63) (number (%))	P value	Mitral regurgitation (n=442) (number (%))	Aortic regurgitation (n=191) (number (%))	P value
Beta-blocker	57 (10)	22 (35)	<0.0001			
Diuretics	511 (90)	39 (60)	<0.0001	375 (85)	153 (80)	0.12
ACE inhibitors/ARBs				88 (20)	47 (25)	0.16
Digoxin	227 (40)	3 (5)	<0.0001			

## Discussion

Most of the patients with rheumatic heart disease with mitral stenosis were on diuretics as most often, they present with shortness of breath secondary to passive pulmonary congestion, and diuretics are the most common drug prescribed by practicing physicians. Of mitral stenosis patients, 90% were on diuretics to relieve dyspnea secondary to pulmonary congestion. General practitioners tend to overprescribe digoxin to patients with rheumatic mitral stenosis as reflected in our study. Of the patients with mitral stenosis, 40% were receiving digoxin therapy in spite of the fact that only 15% of them were having atrial fibrillation. Digoxin has a limited role in patients with mitral stenosis without atrial fibrillation [3]. Only 5% of patients with atrial fibrillation were on verapamil for rate control as it is an uncommon drug used in routine practice. Physicians like to prescribe digoxin for rate control as compared to verapamil as noted in our study. Interestingly, in our study, only 10% of the patients were on beta-blockers, which is the cornerstone in the management of mitral stenosis [4], as beta-blockers, by producing bradycardia, prolong the ventricular diastole, and more blood rushes from the left atrium to the left ventricle and left atrial pressure falls, pulmonary venous congestion decreases, and the patient gets relief from breathlessness. However, physicians were not aware of prescribing beta-blockers as the first-line drug to relieve dyspnea in mitral stenosis. Only 30% of the patients were practicing salt restriction (<5 gm/day), and a very small number of patients (5%) were practicing restricted water intake of less than 1.5 L/day. Restriction of water intake has a promising role in severe mitral stenosis as decreased water intake decreases venous return, decreases left atrial pressure, and decreases passive pulmonary venous congestion.

An interesting observation in patients with rheumatic mitral regurgitation was that most (85%) of patients with rheumatic regurgitation were on diuretics in spite of the fact that diuretics have a limited role in chronic mitral regurgitation without left ventricular systolic dysfunction in whom left atrial pressure rises secondarily to a rise in left ventricular end-diastolic pressure and diuretic therapy decreases left atrial pressure and passive pulmonary venous congestion. Diuretics were overprescribed in patients with chronic rheumatic mitral regurgitation. ACE inhibitors or ARBs decrease the afterload [5], decrease the central aortic pressure, and increase the forward flow during systolic ejection so that less amount of blood regurgitates into the left atrium, thereby decreasing the mitral regurgitation. However, paradoxically, only 20% of the patients with rheumatic mitral regurgitation were on ACE inhibitors or ARBs as noted in our study, which is the cornerstone of therapy in mitral regurgitation.

Diuretics play a detrimental role in patients with aortic stenosis [6] as it decreases the preload for which the force of contraction of the heart decreases, which is the sole criterion to push blood across a stenotic orifice. High-dose diuretic in severe aortic stenosis decreases the cardiac output, blood pressure falls, and the patient may suffer from low cardiac output symptoms in the form of syncope. Strikingly 60% of the patients with severe aortic stenosis were on full doses of diuretics, which should be avoided. If diuretics were prescribed for aortic stenosis, they are prescribed in lower doses, almost in half doses, to decrease venous congestion secondary to an increase in the left ventricular end-diastolic pressure. Beta-blockers are relatively contraindicated in severe aortic stenosis [7] as they also decrease the force of contraction of the heart, thereby decreasing the forward cardiac output in severe aortic stenosis and making the patients suffer from more fatiguability and syncope. However, they were prescribed in a good number of cases, almost one-third of cases, as adjunctive therapy in aortic stenosis with either associated hypertension or episodic palpitation, which may be atrial fibrillation better treated with digoxin. Digoxin has a therapeutic role in severe aortic stenosis as it increases cardiac output by increasing the force of contraction of the heart, but surprisingly, it was prescribed only in 5% of cases of severe aortic stenosis. Severe aortic stenosis in the elderly is associated with coronary artery disease in almost 25% of cases, but in our study, only 10% were on antiplatelets and statin therapy for retarding the progression of valvular lesions [8,9] and prevention of coronary artery diseases in those cohorts of patients.

Diuretics have a limited role in aortic regurgitation unless the patient has left ventricular systolic dysfunction with raised left ventricular systolic pressure and elevated left atrial pressure, where decongestive therapy decompresses the atrium and alleviates the symptoms. However, in our study, diuretics were overprescribed in almost 80% of cases, although only 5% of cases were having left ventricular systolic dysfunction. ACE inhibitors or angiotensin receptor blockers (ARBs) are cornerstones of therapy in the management of aortic regurgitation [10] as they decrease the afterload and decrease the mean aortic pressure, thereby decreasing the extent of regurgitation and total regurgitation volume. Strikingly, only one-fourth of patients with severe aortic regurgitation were prescribed ACE inhibitors or ARBs in severe aortic regurgitation, which is the cornerstone in the management of severe aortic regurgitation. Beta-blockers prolong diastole, thereby increasing regurgitation, and interestingly, 45% of aortic regurgitation patients were on beta-blockers, which would have been wrongly prescribed for pounding sensation/palpitation symptoms in patients with aortic regurgitation secondary to volume overload.

An interesting observation in our study was that 95% of the patients were on oral penicillin prophylaxis (tablet pentid 400 mg, one tablet twice daily), and only 5% of the cases were on proper and recommended injectable benzathine penicillin prophylaxis [11], which may be secondary to the unwillingness of patients to take painful intramuscular penicillin injection and the unwillingness of the peripheral practitioners to administer penicillin in view of possible anaphylaxis. This may be the possible explanation behind the low mean age of the patients with severe rheumatic stenosis where the disease may have progressed exponentially secondary to the lack of proper penicillin prophylaxis.

Our study provides new insight into the drug and dietary patterns of patients with severe rheumatic heart disease at the pretertiary care level among the population in Eastern India. The present study reflects on the lack of proper management: not prescribing the cornerstone drug in each variety of rheumatic heart disease and overprescription of diuretics, digoxin, and beta-blockers, which, if at all will be curtailed, will improve the morbidity and mortality across the spectrum of severe rheumatic heart disease. Strict adherence toward injectable benzathine penicillin prophylaxis could have retarded the disease progression across those populations. Rationale prescription in patients with rheumatic heart disease will alter the natural history and improve the outcome of patients with rheumatic heart disease.

## Conclusions

The present study provides new insight into the pretertiary care level drug and dietary patterns of patients with severe rheumatic valvular heart disease among people of Eastern India. Diuretics were overprescribed across the major spectrum of rheumatic heart disease, including mitral regurgitation, aortic stenosis, and aortic regurgitation. The majority of the patients were lacking the golden drug in management: beta-blockers in mitral stenosis, ACE inhibitors or ARBs in mitral and aortic regurgitation, and digoxin, antiplatelets, and statins in aortic stenosis. The majority of the patients were not on recommended injectable benzathine penicillin prophylaxis. Salt restriction was practiced among only less than one-third of the patients. Management of patients with rheumatic heart disease with the above golden first-line drugs with proper penicillin prophylaxis and judicious use of diuretics can significantly improve morbidity and mortality.
